# Cardiac and skeletal muscle abnormality in taurine transporter-knockout mice

**DOI:** 10.1186/1423-0127-17-S1-S20

**Published:** 2010-08-24

**Authors:** Takashi Ito, Shohei Oishi, Mika Takai, Yasushi Kimura, Yoriko Uozumi, Yasushi Fujio, Stephen W Schaffer, Junichi Azuma

**Affiliations:** 1 Department of Pharmacy, School of Pharmacy, Hyogo University of Health Sciences, Kobe, Japan; 2 Department of Clinical Pharmacology and Pharmacogenomics, Graduate School of Pharmaceutical Sciences, Osaka University, Osaka, Japan; 3 Department of Pharmacology, College of Medicine, University of South Alabama, Mobile, AL, USA

## Abstract

Taurine, a sulfur-containing β-amino acid, is highly contained in heart and skeletal muscle. Taurine has a variety of biological actions, such as ion movement, calcium handling and cytoprotection in the cardiac and skeletal muscles. Meanwhile, taurine deficiency leads various pathologies, including dilated cardiomyopathy, in cat and fox. However, the essential role of taurine depletion on pathogenesis has not been fully clarified. To address the physiological role of taurine in mammalian tissues, *taurine transporter-*(*TauT-*) knockout models were recently generated. TauTKO mice exhibited loss of body weight, abnormal cardiac function and the reduced exercise capacity with tissue taurine depletion. In this chapter, we summarize pathological profile and histological feature of heart and skeletal muscle in TauTKO mice.

## Background

Taurine is a most abundant free amino acid in mammalian tissues with an intracellular concentration of 5-20 µmol/g wet weight [1,2]. A number of evidences revealed that taurine is a cytoprotective agent. Supplementation of taurine is effective to a variety of disorders, such as cardiovascular diseases, skeletal muscle disorders, etc. Meanwhile, taurine deficiency related to some kinds of pathophysiological conditions in cats and foxes, such as dilated cardiomyopathy, retinal degradation and reproduction[3-5].

Taurine transporter (TauT; SLC6a6) is a sodium and chloride ion-dependent transporter, and is expressed ubiquitously in mammalian tissues [6]. Since the capacity to synthesize taurine in most tissues, such as heart and skeletal muscle, is limited, maintenance of the large intracellular taurine pool may depend upon uptake of the amino acid from extracellular space via TauT. This transport process requires the accumulation of taurine against a substantial concentration gradient, as the concentration of taurine is 100 fold less in the plasma (20-100 µM) than in the tissues.

Recently, transgenic mice lacking TauT gene have been generated by two groups [7,8]. A variety of disorders has been reported in various tissues, such as eye, kidney, heart, muscle, etc., accompanied with drastic taurine deficiency in TauTKO mice [7-10]. In this chapter, we report the phenotype of TauTKO mice and discuss the role of taurine deficiency in hearts and skeletal muscles.

## Analysis of taurine transporter knockout mice

In our TauTKO mice, targeting construct for generation of transgenic mice was designed to replace exons 2–4 of the *TauT* gene with a cassette containing neomycin-resistance gene [7]. While a truncated TauT mRNA lacking exon 2-4 was detected in TauTKO tissues, taurine influx was eliminated in the cells isolated from TauTKO mice, indicating loss of taurine transport activity in TauTKO mice.  Tissue taurine level is severely decreased in several tissues. Especially, cardiac taurine could not be detected in TauTKO mice, and skeletal muscle taurine level is decreased by 96% in TauTKO mice compared with wild-type mice. Similarly, in another TauTKO mouse model which was reported by Heller-Stilb et al, is lacking exon 2 of *TauT* gene, and taurine level in skeletal and cardiac muscles was decreased by about 98%, while taurine level in brain, kidney and liver is decreased by 70-90 % compared to wild-type mice [8]. These data illustrate that maintenance of intracellular taurine pool in cardiac and skeletal muscle is extremely dependent upon taurine transport activity.

TauTKO mice exhibited a lower body weight than their control littermates. Furthermore, knocking out TauT causes a decrease in tissue weight, such as heart, skeletal muscle, brain etc [7,8]. Food and water intake were identical in the TauTKO and control mice.

## Cardiac Phenotype of TauTKO mice

Oral supplementation of taurine is effective to animals and human patients with congestive heart failure and cardiomyopathy [11,12], indicating that taurine would play an important role in cardiac homeostasis and cardioprotection against pathological stress. It has been reported that taurine-deficient diet impaired cardiac function and led to dilated cardiomyopathy in cat and fox, which have very low capacity of taurine synthesis [3,13]. Furthermore, drug-induced taurine deficiency by the inhibition of taurine uptake using guanidinoethane sulfonate (GES) or β-alanine led to some cardiac defects in mice or rats [11,14].

In TauTKO model, we determined the cardiac function of TauTKO mice. Echocardiographic analysis revealed that fractional shortening was diminished in the old (>9-month-old) TauTKO mice, whereas we failed to detect a functional difference between the young (5-month-old) wild-type mice and KO mice (Table 1) [7]. Detailed functional analysis on Langendorff perfused heart also demonstrated the age-dependent cardiac dysfunction in TauTKO mice (Unpublished data).

**Table 1:** Echocardiographic analysis of young and old TauTKO mice

	Young (5-month-old)		Old (>9-month-old)	
Genotype	WT (n=3)	TauTKO (n=4)	WT (n=5)	TauTKO (n=7)
LVIDd (cm)	0.37±0.03	0.35±0.03	0.41±0.01	0.40±0.01
LVIDs (cm)	0.25±0.04	0.26±0.02	0.27±0.01	0.31±0.01
FS (%)	30.87±9.22	25.65±4.75	34.18±2.52	22.48±1.92*

LVIDd; left ventricular end-diastolic internal dimension, LVIDs; left ventricular end-systolic internal dimension, FS; fractional shortening, *; p<0.01 v.s. WT.

**Figure 1:**
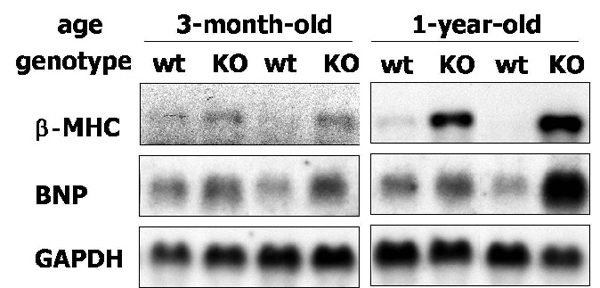
**Age-dependent induction of heart failure markers in TauTKO mice** Northern blot analyses of mRNA from wild-type (WT) and TauTKO (KO) hearts revealed the increased expression of mRNA for brain natriuretic peptide (BNP) and β-myosin heavy chain (βMHC) in TauTKO mice. GAPDH mRNA was measured to internal control.

It is well established that the expression of fetal genes, including atrial natriuretic peptide (ANP), brain natriuretic peptide (BNP) and β-myosin heavy chain (βMHC), is reactivated in a variety of cardiac pathological conditions, such as ischemia, heart failure, hypertrophy and atrophy [15]. These cardiac failure markers were significantly elevated in both young and old TauTKO mice (Fig. 1) [7]. Consistent with the cardiac function, the inductions of these genes were more significant in the old TauTKO mice. These data indicate that knockout of the TauT gene leads to an age-dependent dilated cardiomyopathy.

On the other hand, Warskulat et al. reported that TauTKO mice exhibited normal cardiac function [9]. These inconsistent results between two TauTKO models may be due to the difference of genetic background, since the difference of inbred strains affects the cardiovascular phenotypes and susceptibilities against pathological stressors in mice [16]. We used mice which backcrossed at least 4 times into C57BL/6 line to minimize genetic differences, while Warskulat et al. reported the use of F2 mixed C57BL/6 amd 129/SvJ strains. Meanwhile, Warskulat et al. also reported that biomarker genes for heart failure, including ANP, BNP and CARP, are upregulated in TauTKO hearts consistent with our TauTKO model [17]. Furthermore, they also demonstrated that the TauTKO hearts showed a switch from alpha-actin 1 (skeletal muscle type) to alpha-actin 2 (smooth muscle type) expression [17]. These data suggest that other TauTKO mice (Warskulat et al) may have the aptitude toward heart failure, and hearts may be more susceptible to exogenous stresses in TauTKO mice.

Histological analysis revealed that TauTKO mice undergo ventricular remodeling, characterized by dilated ventricles and reductions in ventricular wall thickness [7]. Furthermore, cross sectional area of ventricular cardiomyocytes was decreased in TauTKO hearts, implying the importance of taurine for cell size. Surprisingly, cardiac fibrosis was not observed in TauTKO heart. Transition electron microscopic analysis demonstrated that TauTKO hearts exhibited significant ultrastructural damage of myofilament and mitochondria. Furthermore, another important feature of TauTKO mice is the presence of autophagosome containing mitochondria. Autophagy is a biological process, in which cells degrade and recycle intracellular macromolecules and organelles. It is considered to represent a cellular adaptation to ensure survival, as injured and potentially damaging organelles are targeted for elimination. There are several triggers of mitochondrial autophagy, such as mtDNA damage, lipid peroxidation and the mitochondrial permeability transition [18-20]. Thus, the triggers themselves might provide useful information on the pathology that is occurring in the taurine knockout heart.

## Phenotype of exercise capacity and skeletal muscle

Taurine is well known to modulate ion movement and play a role in the excitation-contraction coupling mechanism in skeletal muscle [21], 22. It has been reported that supplementation of taurine improved exercise capacity in rats and attenuated the exercise-induced oxidative injury [23,24]. However, the effect of taurine deficiency on the exercise has been unclear. It has been reported that supplementation of GES to decrease in taurine content in muscles reduced force output and increased the endurance of skeletal muscles in rats [25].  However, since GES itself directly increases susceptibility to Ca^2+^ on isolated muscle skinned fiber, the pharmacological action of GES must influence to the experiments using taurine depleted animal models [26].

In TauTKO mice, weight-loaded swimming test revealed that exercise endurance time was severely reduced compared to wild-type mice (118 ± 2.3 min in wild-type vs 10 ±2.5 min in TauTKO, p<0.01) [7]. Additionally, forced treadmill test on uphill road also revealed that the duration of running time to exhaustion is reduced in TauTKO mice (49.0±11.0 min in wild-type vs 14.8±9.2 min in TauTKO, p<0.01). Warskulat et al. have also reported that total running distance to exhaustion on the treadmill is reduced by more than 80% in TauTKO mice [9]. These data indicate that taurine deficiency may reduce muscle function in skeletal muscles. Moreover, Warskulat et al. demonstrated that X-ray studies of the skeleton did not reveal morphological disorders in TauTKO mice, indicating that skeletal muscle abnormalities may be associated with the reduction of exercise capacity in TauTKO mice.

**Figure 2:**
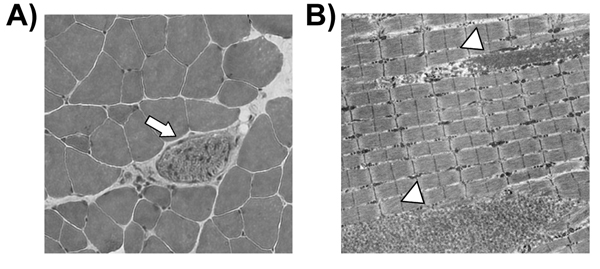
**Histological disorders in skeletal muscle of TauTKO mice.** A) Representative micrographs of tibial anterior muscle sections of TauTKO mice. The arrow indicates a necrotic cell. B) Representative electron micrographs of tibial anterior muscle sections of TauTKO mice. Magnification; ×3000. The arrowheads indicate myofilament fragmentations.

Since TauTKO mice lost muscle mass, the skeletal muscle was analyzed histologically [7]. As expected for atrophic muscle, the myofibrillar cross sectional area of the tibial anterior muscle of the mutant mice was remarkably reduced compared to that of their littermate controls. Furthermore, necrotic cells were detected in TauTKO muscles (Fig. 2A), indicating that taurine may play a role in the regulation of cell volume and cell survival in skeletal muscle cells. Electron microscopy revealed some kinds of ultrastructural abnormalities in TauTKO muscle, such as filament fragmentation, membranous cytoplasmic body and lipid droplet (Fig. 2B). These observations suggest that taurine depletion may result in destabilization of myofilament and other cytosolic organelle in skeletal muscle. Clearly, further studies are required to clarify the mechanism underlying taurine depletion-induced muscle disorders.

## Conclusion

TauTKO mice displayed the dilated cardiomyopathy, consistent with the phenotype of taurine-depleted cats. These data illustrate that taurine depletion is an independent etiology of cardiomyopathy. This model will provide benefits to find the molecular mechanism underlying taurine-depleted cardiomyopathy. Furthermore, lacking TauT results in aging-dependent cardiac dysfunction, indicating that taurine deficient hearts might be less able to tolerate aging. Aging-dependent disorders have been reported in several tissues of TauTKO mice, including visual, auditory, olfactory and renal dysfunctions and hepatitis [10,27,28]. Taurine deficiency i likely to increase susceptibility against stress, such as oxidative stress, which in turn causes to accelerated aging.

## Competing interests

The authors declare that they have no competing interests.
